# Phenotypic and genotypic analysis of *Acinetobacter baumannii* isolated from combat wounds in Ukraine during 2022 and 2023

**DOI:** 10.1093/jacamr/dlaf140

**Published:** 2025-08-07

**Authors:** V M Kondratiuk, Brendan T Jones, Ting L Luo, N S Fomina, Francois Lebreton, Jason W Bennett, Patrick Mc Gann, V P Kovalchuk

**Affiliations:** Department of Emergency and Military Medicine, National Pirogov Memorial Medical University, Vinnytsia, Ukraine; Multidrug-Resistant Organism Repository and Surveillance Network (MRSN), Bacterial Diseases Branch, CIDR, Walter Reed Army Institute of Research, Silver Spring, MD, USA; Multidrug-Resistant Organism Repository and Surveillance Network (MRSN), Bacterial Diseases Branch, CIDR, Walter Reed Army Institute of Research, Silver Spring, MD, USA; Department of Microbiology, National Pirogov Memorial Medical University, Vinnytsia, Ukraine; Multidrug-Resistant Organism Repository and Surveillance Network (MRSN), Bacterial Diseases Branch, CIDR, Walter Reed Army Institute of Research, Silver Spring, MD, USA; Multidrug-Resistant Organism Repository and Surveillance Network (MRSN), Bacterial Diseases Branch, CIDR, Walter Reed Army Institute of Research, Silver Spring, MD, USA; Multidrug-Resistant Organism Repository and Surveillance Network (MRSN), Bacterial Diseases Branch, CIDR, Walter Reed Army Institute of Research, Silver Spring, MD, USA; Department of Emergency and Military Medicine, National Pirogov Memorial Medical University, Vinnytsia, Ukraine

## Abstract

**Background:**

*Acinetobacter baumannii* is an important nosocomial pathogen worldwide. During the current invasion of Ukraine, reports of infections caused by this organism have proliferated. Here, we provide a phenotypic and genotypic analysis of *A. baumannii* associated with the conflict.

**Methods:**

Between March 2022 and September 2023, 68 *A. baumannii* strains were cultured from wounded Ukrainian service members in three hospitals in west-central Ukraine. Antibiotic susceptibility and WGS were performed on all isolates.

**Results:**

Strains encompassed eight different STs, including the emerging ST78 (and its single locus variant ST1077) and globally distributed ST2 lineages, with ST19 being the most common (25%). Fifty strains carried at least one acquired carbapenemase (*bla*_OXA-23_ or *bla*_OXA-72_), with seven strains carrying both. Overall, susceptibility ranged from 0% (fluoroquinolones) to 100% (SUL/durlobactam) and all strains had CST MICs <1 mg/mL. Notably, all but one ST2 isolates were resistant to FDC, and this correlated with the presence of the *bla*_PER-1_ or *bla*_PER-7_ ESBL genes. In contrast, 8 of 13 ST78 were FDC non-susceptible, but non-susceptibility was correlated with the disruption of the *pirA* siderophore receptor gene by IS*Aba35*. Finally, passage in MEM of one strain for 8 days resulted in a mutation of the *bla*_GES-11_ ESBL to the *bla*_GES-14_ carbapenemase.

**Conclusions:**

Sampling of *A. baumannii* strains infecting injured Ukrainian soldiers revealed the predominance of known (ST2) and emerging (ST19, ST78) lineages carrying carbapenemases. Antibiotic resistance was broad, including the recently approved catechol-substituted siderophore cephalosporin, FDC, highlighting the immense treatment challenges faced by medical personnel during this ongoing conflict.

## Introduction

Despite their widespread presence in the environment, bacteria from the genus *Acinetobacter* were largely absent from medical studies until the 1980s, when multiple reports highlighting their prevalence in hospitals and their potential to cause significant medical problems began to emerge.^1^ The role of *Acinetobacter baumannii* as a nosocomial pathogen became more evident during the Iraq and Afghanistan conflicts, with numerous infections in wounded patients treated in military facilities.^2^ Notably, these pathogens exhibited high antimicrobial resistance, including the emergence of carbapenem-resistant strains.^3^ Since then, *A. baumannii* has been identified as a global problem,^4^ and carbapenem-resistant *A. baumannii* has been designated as a critical pathogen for the research and development of new antibiotics by the WHO (https://www.who.int/publications/i/item/9789240093461; last accessed February 2025).

After the start of the war in eastern Ukraine in 2014, *A. baumannii* became a common pathogen cultured from combat wounds, with most isolates being multidrug resistant (MDR).^5,6^ A subsequent genetic analysis of these isolates revealed they belonged to five different STs, including global ST1 and ST2 carrying a variety of Class D carbapenemases, as well as more regional strains such as ST19, ST78 and ST400.^7^ Following the full-scale invasion by Russia in 2022, the significant increase in casualties once again prompted a rise in *A. baumannii* wound infections in Ukraine hospitals. In parallel, reports of *A. baumannii* being cultured from Ukrainian patients admitted to European hospitals were also published, including strains belonging to ST2 and ST78.^8–12^

We recently reported on the temporal evolution of bacterial species from war-related injuries between 2014 and 2023.^13^ In this study, we provide a subsequent comprehensive genomic and phenotypic analysis of the 68 *A. baumannii* strains, including their susceptibility to newer antimicrobials.

## Methods

### Bacteriological methods

From March 2022 to September 2023, bacteriological swabs were taken from combat-related injuries of 171 patients who were treated at three definitive care hospitals (NATO ROLE III-IV) in central Ukraine. The collection of wound exudate was performed using the BD BBL Culture Swab Plus collection and transport swabs (Becton Dickson, USA). Pure cultures were isolated from the swabs using two nutrient media: tryptic soy agar and chromogenic agar for *Acinetobacter* (Graso Biotech, Poland). The sampling included male soldiers with evident signs of surgical wound infection from whom swabs were collected within 12 h of admission during their initial surgical examination with wound bandage opening. Only extremity wounds were included in the sampling. On average, these individuals had been injured 6.2 ± 3.9 days prior to swabbing and had been transferred through four to five evacuation hospitals ranging from NATO Level II to III before arriving at the final facility.

In total 68 strains of *A. baumannii* bacteria were isolated and identified (Table S1, available as Supplementary data at *JAC-AMR* Online). Duplicate strains from the same infection source were excluded. Antimicrobial susceptibility testing (AST) was initially performed in hospital laboratories via the disk diffusion method according to the EUCAST with Updates and Supplements.

### Additional phenotypic characterization of isolates

All isolates were sent to the Multidrug-Resistant Organism Repository and Surveillance Network (MRSN) at the Walter Reed Army Institute of Research in the USA, where they underwent additional AST on the Vitek2 with Cards N808 and XN-32. MICs of FDC (Shionogi Pharma, Japan) and SUL-durlobactam (SUL/DUR, Entasis Therapeutics Inc., USA) were determined in triplicate using broth microdilution (BMD) with CLSI guidelines^14^ at concentrations ranging from ≤0.25 to 128 mg/L for FDC and ≤0.125 to 64 mg/L for SUL/DUR (with DUR at a fix concentration of 4 mg/L). CST MICs were determined in triplicate using the Sensititre Automated AST System (Thermo Fisher Scientific, MA, USA) and a customized Sensititre plate with concentrations ranging from 0.25 to 16 mg/L. Breakpoints were determined using CLSI recommendations.^15^ Strains were defined as MDR or XDR using a modification of the definitions proposed by Magiorakos *et al*.^16^ based on the 14 antibiotics (from 11 categories) tested (Table S1). Isolates non-susceptible to ≥1 agent in ≥3 of the 11 antimicrobial categories were classified as MDR and isolates non-susceptible to ≥1 agent in at least 8 of the 11 antimicrobial categories were classified as XDR.

### WGS, core genome MLST, SNP calling and phylogenetic analysis

WGS of all isolates was performed on an Illumina MiSeq benchtop instrument as previously described.^17^ Briefly, DNA was extracted using the DNeasy UltraClean Microbial Kit (Qiagen, Germantown, MD, USA) and libraries were constructed using the KAPA HyperPlus Library preparation kit (Roche Diagnostics, Indianapolis, IN, USA). Libraries were quantified using the KAPA Library Quantification Kit—Illumina/Bio-Rad iCycler^™^ (Roche Diagnostics) on a CFX96 real-time cycler (Bio-Rad, Hercules, CA, USA). Libraries were normalized to 2 nM, pooled, denatured and diluted to a final concentration of 16 pM.

Genomes were sequenced using an Illumina MiSeq platform with the MiSeq Reagent Kit v3 (600 cycles; 2 × 300 bp). Kraken2 v2.1.2^18^ was used for initial taxonomic assignment and to screen for contamination. *De novo* draft genome assemblies were produced using Shovill v1.1.0 (https://github.com/tseemann/shovill) with coverage estimates generated using BBmap v38.96. Minimum thresholds for contig size and coverage were set at 200 bp and 49.5+, respectively. In cases where the Kraken2-derived taxonomic assignment was ambiguous, the Genome Taxonomy Database (GTDB)^19^ was used via the GTDB-Tk v2.4.0.^20^ Genomes were annotated using Bakta v.1.10.4 (https://github.com/oschwengers/bakta). Antimicrobial resistance genes were annotated using a combination of ARIBA v2.14.6^21^ and AMRFinderPlus v3.12.8).^22^ MLST assignment was performed using mlst v2.22.1 (https://github.com/tseemann/mlst) using the Pasteur scheme.^23^

Finally, genome assemblies were used as input for Roary v3.13.0 (https://sanger-pathogens.github.io/Roary/) and an SNP-based alignment of core genes was generated. Recombination was filtered from the alignment using Gubbins v2.4.1 (https://github.com/nickjcroucher/gubbins) and a maximum-likelihood tree was generated with RAxML-NG (v1.1) (https://github.com/amkozlov/raxml-ng) using the GTR+G (50 parsimony, 50 random) model 100 random bootstrap replicates. The tree was imported in iTOL (v6.8.1)^24^ for visualization with metadata.

### Serial passage of *A. baumannii* in the presence of MEM

MRSN 122177, a carbapenemase-negative but MEM intermediate strain was selected for further analysis. The strain was initially grown overnight in 20 mL of Mueller–Hinton broth (MHB) at 37°C with shaking in 50 mL conical tubes with MEM at a final concentration of 2 mg/L. Of the resulting growth, 100 µL was then used to inoculate fresh MHB and the *A. baumannii* serially passaged in increasing two-fold concentrations of MEM to 32 mg/L over a period of 8 days. No further growth was recorded at higher concentrations and the resulting strain was analysed using WGS.

## Results and discussion

### Phenotypic characterization of isolates

**Figure 1.**
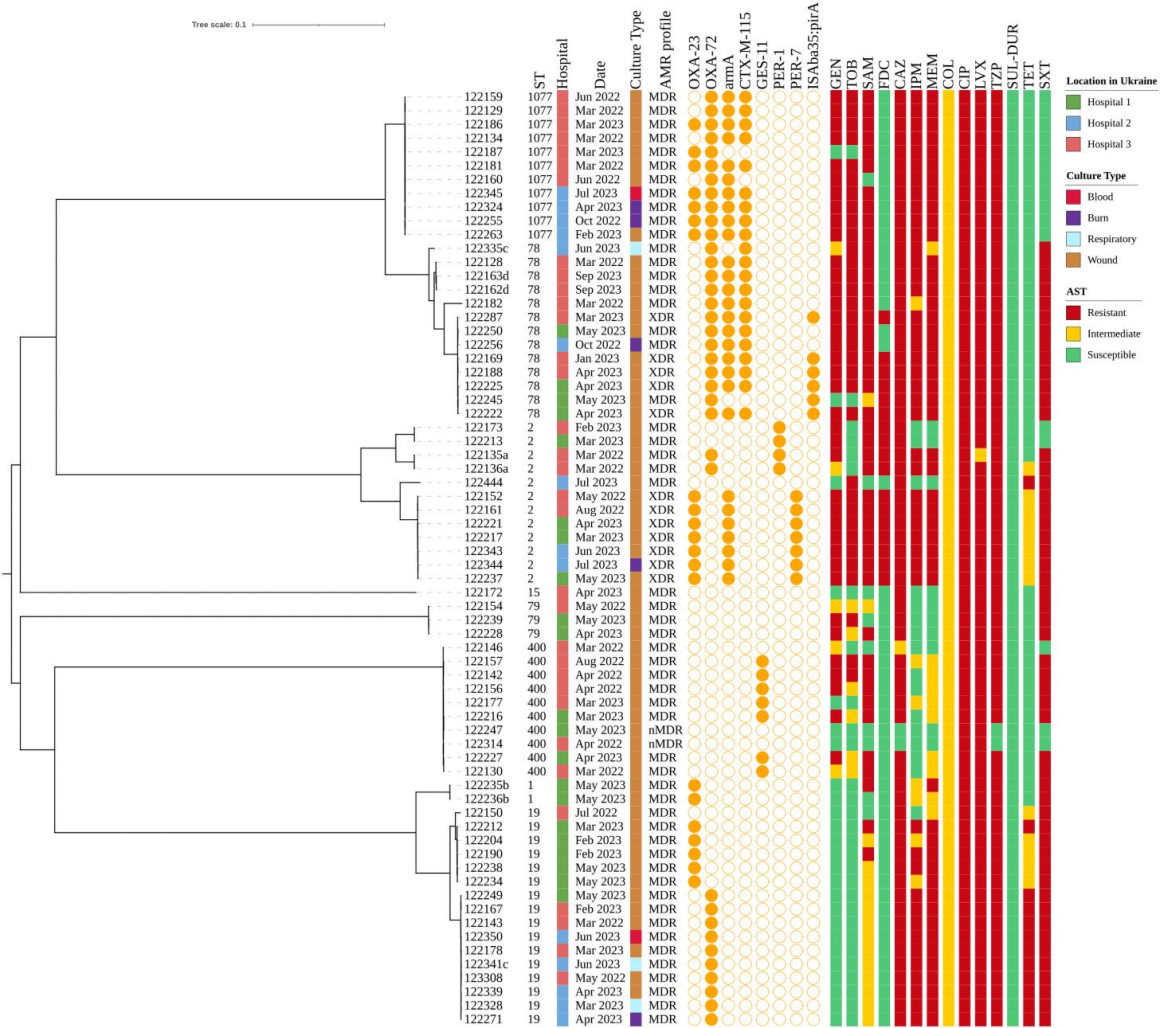
Midpoint-rooted, maximum-likelihood phylogenetic tree based on the core genome of 68 *A. baumannii* strains collected from three hospitals in Ukraine, between March 2022 and September 2023. ST, hospital of origin, date of collection, culture type, and presence (closed symbol) or absence (open symbol) of selected antimicrobial resistance genes (circle) and phenotypic resistance to select antibiotics (square) are indicated. Serial isolates (same patients) are indicated with superscript letters next to the isolate name.

Of the 68 of *A. baumannii* strains in this study, 55 (95.6%) were MDR, with 12 (17.6%) also being classified as XDR (Figure 1). Notably, all isolates were non-susceptible to the fluoroquinolones, with 97.1%, 86.7% and 76.4% also being resistant to TZP, SAM and SXT, respectively. Similarly high levels of resistance were noted for the aminoglycosides (∼60%) and the β-lactams, including the carbapenems (76.5%). Notably, 25% were also resistant to the recently approved catechol-substituted siderophore cephalosporin, FDC (see below for details).^25^ In contrast, only two antibiotics demonstrated broad activity, CST and the recently approved combination therapy SUL/DUR (Table S1).^26^

### Two emerging lineages of *A. baumannii* ST19

*A. baumannii* ST19 was the largest clonal group identified in this study, comprising 23.5% of all isolates cultured. Fifteen of 16 isolates carried a Class D carbapenemase, either *bla*_OXA-23_ (*n* = 5) or *bla*_OXA-72_ (*n* = 10) (Figure 1). We recently showed that *A. baumannii* ST19 carrying *bla*_OXA-23_ form a distinct sub-lineage to those with *bla*_OXA-72_ and that both lineages have recently emerged in Georgia and Ukraine.^27^ In that study, long-read sequencing revealed that *bla*_OXA-72_ was encoded on an R3-type plasmid, while two identical copies of *bla*_OXA-23_ were found, one on an RP-type plasmid and the other in the chromosome.^27^ In this study, all isolates carrying *bla*_OXA-23_ were only cultured from patients receiving care in Hospital 1 (Figure 1), while all but one (MRSN 122249) carrying *bla*_OXA-72_ were cultured from patients in Hospitals 2 and 3. All ST19 isolates were susceptible to CST (MICs < 1 mg/L), FDC (MICs ≤ 4 mg/L) and the aminoglycosides, GEN and TOB (<4 mg/L).

### Clonal expansion of *A. baumannii* ST78 and its single locus variant ST1077

Thirteen isolates belonged to ST78 and a further 11 belonged to its single locus variant (SLV), ST1077. All 11 ST1077 isolates formed a cluster of highly genetically related isolates (distinct by only 0–18 SNPs) that formed a distinct branch with the more genetically diverse ST78 (Figure 1). All isolates in both clonal groups carried the *bla*_OXA-72_ carbapenemase, but seven ST1077 isolates had also acquired *bla*_OXA-23_. In addition, all but two isolates carried the *armA* 16S methyltransferase gene, and this clade also exclusively carried the ESBL gene *bla*_CTX-M-115_, a hallmark of the ST78 clade.^28^ Though both ST78 and ST1077 displayed both MDR and XDR phenotypes, the exclusive resistance to trimethoprim/sulfamethoxazole in the ST78 isolates can be attributed to the presence of *sul2*, which is absent in the ST1077 isolates. All ST78 isolates were susceptible to CST and SUL/DUR but just 5 of the 13 isolates were susceptible to FDC (MICs, Figure 1). A comprehensive analysis of the genomes revealed that the *pirA* gene in all eight FDC resistant isolates was disrupted by an IS, predicted to result in a non-functional PirA siderophore receptor. In contrast, the gene was intact in all five FDC-susceptible isolates (Figure 1). Disruption of *pirA* by IS*Aba35*, and truncation of PirA due to *pirA* mutations, have previously been shown to confer FDC resistance in *A. baumannii*.^29,30^ In contrast, all 13 isolates had identical *pbp3, piuA, baeR* and *baeS* genes, indicating that FDC resistance in this clade was likely mediated via loss of the PirA receptor.

ST78 belongs to International Clone 6 (IC6) and have acquired the moniker ‘The Italian clone’ due to their first being isolated there in the mid-2000s.^31^ Since then, the lineage has been sporadically described in Europe,^32,33^ though an association with patients treated in Russia has also been reported.^28,34^ More recently, ST78 and ST1077 have been routinely cultured from injured Ukrainian service members both before^5,7^ and after^13^ the full-scale invasion in 2022, as well as among Ukrainian patients receiving care in hospitals in Germany and Denmark.^11,35^ These reports and our data suggest that this lineage and its emerging SLV ST1077 is likely endemic in Ukraine and further surveillance for this emerging clonal group is warranted.

### Inter-hospital spread of global clone ST2

Twelve isolates from 11 patients were assigned to ST2, the most common lineage of *A. baumannii* worldwide and strongly associated with carbapenemase production.^36^ In agreement, 9 of the 12 isolates carried a carbapenemase, either *bla*_OXA-23_ (*n* = 7) or *bla*_OXA-72_ (*n* = 2) (Figure 1). Overall, the isolates could be separated into two sub-lineages: one comprising four *bla*_PER-1-_carrying strains, two of which also carried *bla*_OXA-72_, and another sub-lineage comprising the seven *bla*_OXA-23_-containing strains (Figure 1) in addition to *armA* and the ESBL *bla*_PER-7_. Notably, the latter group consisted of highly genetically related isolates (distinct by only 1–14 SNPs) cultured from patients across all three hospitals between May 2022 and July 2023, suggesting that this lineage may be endemic in this area.

All isolates were susceptible to CST and SUL/DUR, but 11 of the 12 isolates were resistant to FDC, with MICs of 64 and 128 mg/L for all 11 isolates. Recently, Poirel *et al*. have shown that the presence of PER enzymes, and to a lesser extent NDM, results in high level FDC resistance in *A. baumannii*, and the presence of PER-1 and PER-7 in our strains likely accounts for the observed FDC resistance.^37^ In support of this, MRSN 122444 is the only isolate not carrying a *bla*_PER_ gene and is susceptible to FDC (Figure 1; MIC of 0.5 mg/L) and the *pbp3, piuA, pirA, baeR* and *baeS* genes are identical in all strains.

### Conversion of GES11 (ESBL) to GES14 (carbapenemase) in ST400

Ten *A. baumannii* strains belonging to ST400 were recovered from 10 patients across two hospitals between March 2022 and May 2023, and none carried a carbapenemase (Figure 1). Three pairs of isolates were nearly genetically identical (MRSN 122142 and −122157 from hospital 3 distinct by 11 SNPs, MRSN 122227 and −122130 from Hospitals 1 and 3 distinct by nine SNPs; and MRSN 122247 and −122216 from Hospital 1 distinct by only six SNPs), suggestive of recent nosocomial transmission events. The remaining isolates were more distantly genetically related (distinct by 58–114 SNPs), suggestive of a more ancient common ancestor in Ukraine hospitals. Seven isolates carried *bla*_GES-11_, a variant of the ESBL *bla*_GES-1_ gene that confers the ESBL phenotype and also reduced susceptibility to carbapenems.^38^ This was confirmed by antibiotic susceptibility testing, which showed that all GES-carrying isolates had elevated MICs to IPM compared with their GES-negative relatives (MIC of 1–2 compared with <0.25 mg/L), and that only GES-carrying isolates had intermediate susceptibility to MEM (MIC of 4 compared with 0.5 mg/L).

Because all GES-carrying ST400 isolates had intermediate resistance to MEM, we wanted to examine the effects of MEM exposure on MICs. Previous studies have shown that related strains of *A. baumannii* from Turkey, Gaza and Egypt carried either GES-11 and GES-14 (a single loci variant of GES-11 due to a Gly170Ser substitution) on the same Class 1 integron structure, suggesting a common ancestor.^39,40^ Therefore, we selected the MRSN 122177 strain, an ST400 isolate that carried the *bla*_GES-11_ ESBL, for passage on increasing concentrations of MEM. The strain was initially intermediate to MEM (MIC = 4 mg/L) and after passage for 8 days on increasing concentrations of MEM, the MICs had increased to 32 mg/L, with a corresponding increase in IPM MICs from 2 mg/L (susceptible) to >32 mg/L (resistant). WGS revealed that increased resistance to both carbapenems could be traced to a single non-synonymous point mutation within *bla*_GES-11_ that resulted in the canonical Gly170Ser substitution resulting in the GES-14 carbapenemase.^40^

Previously, ST400 has been cultured from Ukrainian service members injured in eastern Ukraine prior to the 2022 invasion, but none of the isolates from that study carried *bla*_GES_.^7^ More recently, Fursova *et al*. described an ST400 strain cultured in 2018 from the glioma of a 58-year-old woman at a Moscow neurosurgery ICU.^34^ However, unlike the strains in this study, their isolate carried *bla*_PER-1_ and *bla*_GES-1_. Other sporadic detections of this clone have been described in Germany^41^ and Brazil,^42^ but the true distribution of this emerging clone remains obscure.

### Sporadic detection of other *A. baumannii* lineages

Six additional isolates from five patients were also cultured during the period of this study. Two ST1 isolates carrying the *bla*_OXA-23_ carbapenemase were cultured from the same patient at Hospital 1 in May 2023, and both isolates were genetically identical. Similarly, in April 2023, a single ST15 isolate carrying no major antibiotic resistance genes was cultured from a patient in Hospital 3. Finally, three ST79 isolates were cultured from the war wounds of three separate patients: one in Hospital 3 in May 2022 and two from patients in Hospital 1 in April and May 2023 (Figure 1).

### Conclusions

In the context of traumatic epidemics, such as wars, additional favourable conditions for the proliferation of MDR organisms are created, which is confirmed by our results. In our study, all but two isolates were MDR, almost triple the rates observed early in the Iraq and Afghanistan conflicts.^43^ This disappointing dynamic can be explained by the force majeure of the medical care system in a large-scale war, which complicates the implementation of infection control measures.^44^ Carbapenemase-producing *A. baumannii*, particularly those carrying *bla*_OXA-23_ and *bla*_OXA-72_, were found across all three hospitals, with just CST and SUL/DUR demonstrating broad coverage. The ultimate source of these isolates remains to be determined, but lessons learned during the Iraq and Afghanistan conflicts have emphasized the essential need for robust surveillance and screening of patients upon admittance to hospitals.^45^ Studies during these conflicts indicated that it was unlikely that wounds were being colonized at the time of injury due to the lack of MDR bacteria upon initial trauma.^46,47^ Instead, it has been postulated that nosocomial transmission was responsible,^48^ as recently confirmed for a clone of ST1 *A. baumannii* cultured from 30 patients at a major US military hospital during this period.^49^

Controlling and preventing the spread of these pathogens is challenging, but an aggressive approach that included hand hygiene, contact barrier precautions, patient and staff cohorting, chlorhexidine oral care, reducing the duration and spectrum of surgical antimicrobial prophylaxis, education and command emphasis were instrumental in reducing the rates of ventilator-associated pneumonia (VAP) rate (VAP per 1000 ventilator-days) from 60.6 to 11 in just 6 months.^50^ Adherence to these strict protocols, combined with continued monitoring and surveillance, are essential to understand the transmission of these pathogens better and implement effective controls to prevent their spread.

## Supplementary Material

dlaf140_Supplementary_Data

## Data Availability

Genomic assemblies of all isolates analysed in this study are publicly available in the NCBI database under the BioProject numbers PRJNA1101874 and PRJNA1162747. The genome of MRSN 144391, the derivative of MRSN 122177 that acquired the Gly170Ser conversion from *bla*_GES-11_ to *bla*_GES-14_, has been deposited at NCBI under BioSample SAMN48893096.

## Supplementary data

Table S1 is available as Supplementary data at *JAC-AMR*  Online.
